# The effect of body mass index on smoking behaviour and nicotine metabolism: a Mendelian randomization study

**DOI:** 10.1093/hmg/ddy434

**Published:** 2018-12-18

**Authors:** Amy E Taylor, Rebecca C Richmond, Teemu Palviainen, Anu Loukola, Robyn E Wootton, Jaakko Kaprio, Caroline L Relton, George Davey Smith, Marcus R Munafò

**Affiliations:** 1 Population Health Sciences, Bristol Medical School, University of Bristol, Bristol, UK; 2 National Institute for Health Research Bristol Biomedical Research Centre, University Hospitals Bristol NHS Foundation Trust and the University of Bristol, UK; 3 MRC Medical Research Council Integrative Epidemiology Unit, University of Bristol, Bristol, UK; 4 Institute for Molecular Medicine Finland FIMM, Helsinki Institute for Life Science, University of Helsinki, Helsinki, Finland; 5 Department of Public Health, Medical Faculty, University of Helsinki, Helsinki, Finland; 6 UK Centre for Tobacco and Alcohol Studies, School of Experimental Psychology, University of Bristol, Bristol, UK

## Abstract

Given clear evidence that smoking lowers weight, it is possible that individuals with higher body mass index (BMI) smoke in order to lose or maintain their weight. We performed Mendelian randomization (MR) analyses of the effects of BMI on smoking behaviour in UK Biobank and the Tobacco and Genetics Consortium genome-wide association study (GWAS), on cotinine levels and nicotine metabolite ratio (NMR) in published GWAS and on DNA methylation in the Avon Longitudinal Study of Parents and Children. Our results indicate that higher BMI causally influences lifetime smoking, smoking initiation, smoking heaviness and also DNA methylation at the aryl-hydrocarbon receptor repressor (*AHRR)* locus, but we do not see evidence for an effect on smoking cessation. While there is no strong evidence that BMI causally influences cotinine levels, suggestive evidence for a negative causal influence on NMR may explain this. There is a causal effect of BMI on smoking, but the relationship is likely to be complex due to opposing effects on behaviour and metabolism.

## Introduction

Smoking and obesity are amongst the leading preventable causes of mortality and morbidity worldwide (1). Understanding pathways that contribute to these risk factors, and the nature of the relationship between them, is therefore of paramount importance for disease prevention. Observationally, current smoking is often associated with lower body mass index (BMI) (2). However, heavy smoking has been found to be associated with higher BMI (2,3). Given the clustering of unhealthy behaviours such as smoking, low physical activity and poor diet (4) and the strong links between smoking, obesity and sociodemographic factors (5), establishing the existence of and direction of causality is difficult.

Mendelian randomization (MR), which uses genetic variants associated with exposures as proxies, can help to overcome problems of confounding and reverse causality because, in theory, genetic variants associated with the exposure of interest should be inherited independently of other genetic variants and environmental factors (6). There is good evidence from MR studies, using a genetic variant that influences the number of cigarettes consumed per day among smokers, that heavier smoking causes a reduction in BMI and other measures of adiposity (7–9). This may be explained by nicotine increasing metabolic rate and/or lowering appetite and therefore changing energy balance (2). To support this, there is a large body of evidence showing that smoking cessation is accompanied by weight gain (10–15), though with large individual variation in the amount gained.

Given that smoking lowers body weight, it is plausible that the association between BMI and smoking is bidirectional; that is more overweight individuals may take up smoking, smoke more heavily or continue to smoke rather than quit, in order to lower weight. Weight gain is commonly cited as a concern for smokers who are considering quitting smoking (10). This has been found most consistently in women (10), although there is also evidence that weight concern is associated with motivation to quit smoking in men (16). Weight concern or body dissatisfaction amongst adolescents may also increase the likelihood of smoking initiation (17,18). However, it is important to note that the relationship between weight concern and BMI is complex; for example, it may be U-shaped in males (19). Amongst young people in the Avon Longitudinal Study of Parents and Children (ALSPAC), higher BMI was associated with smoking initiation in females, but not in males, whereas body dissatisfaction was associated with higher risk of smoking initiation in both sexes (20). Smoking and obesity are also both associated with increased risk of anxiety and depression (21), and there is evidence that the link between higher BMI and depressive symptoms is causal (22). Therefore, it is possible that BMI could lead to smoking through its effects on mental health, although strong evidence of causality between mental health and smoking is yet to be established.

In addition to behavioural links, it is possible that BMI could alter smoking behaviour via physiological effects. Higher BMI could result in lower blood nicotine levels for the same amount smoked, due to higher total blood volume or absorption of nicotine or its metabolites by fatty tissue (23). It has been demonstrated that BMI is negatively correlated with nicotine levels following administration of nicotine replacement therapy (24). This could mean that individuals with higher BMI would need to smoke more in order to experience the same effect of nicotine. BMI may also affect nicotine metabolism, which is commonly measured by the nicotine metabolite ratio (NMR). Studies have shown that individuals with higher NMR (reflecting faster metabolism of nicotine) smoke more heavily and are less likely to give up smoking (25,26). Observationally, BMI tends to be negatively correlated with NMR (27). This could plausibly be because NMR lowers BMI through its effect on increasing smoking, although it has been argued that evidence points towards the relationship being in the opposite direction, from BMI to NMR (27).

A previous genetic analysis demonstrated that higher genetically determined BMI was associated with increased likelihood of smoking initiation and higher tobacco consumption (28). This was interpreted by the authors as shared genetic aetiology for BMI and smoking rather than a causal effect of BMI on smoking. For example, variants in the brain-derived neurotrophic factor (*BDNF*) gene associate with both BMI and smoking initiation at genome-wide significance level (29,30). A more recent MR analysis provided evidence that BMI causally influences smoking behaviour, but was restricted to discrete self-report smoking phenotypes (e.g. initiation, heaviness of smoking) (31).

We sought to extend this work and explore the potential causal effect of BMI on smoking using a larger number of genetic variants and MR methods, which are more robust to potential pleiotropy (32–34). Using genetic variants associated with BMI from the largest published genome-wide association studies (GWAS) of BMI to date (30), we investigated whether BMI causes differences in smoking behaviour and total tobacco exposure by looking at both self-reported measures of smoking and biological measures of exposure (cotinine and DNA methylation). Our self-report measures included individual smoking phenotypes and a composite measure of lifetime smoking. We also used this approach to investigate whether BMI causally influences NMR. We performed analyses using several data sets: the Tobacco and Genetics (TAG) Consortium GWAS (29), the Cotinine Consortium GWAS (35) and the largest NMR GWAS conducted to date (36), the UK Biobank (37) and ALSPAC (38).

## Results

### Association of BMI genetic risk score with BMI

Within UK Biobank, each SD increase in genetic risk score was associated with a 0.64 kg/m^2^ increase in BMI (95% confidence interval (CI): 0.62–0.65). There was evidence that the association of the BMI genetic risk differed by smoking status (*P* for heterogeneity ≤0.001), with the strongest association seen in current smokers (Supplementary Material, Fig. S3).

### MR analysis of effect of BMI on self-reported smoking behaviours

**Figure 1:**
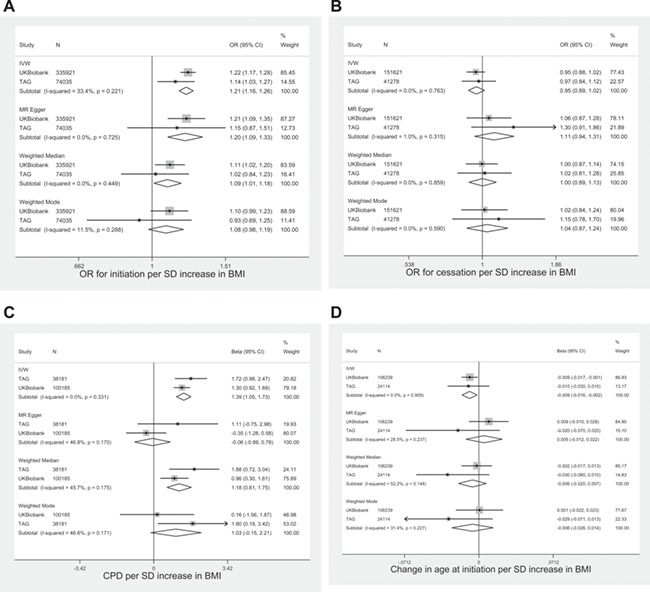
Association between BMI genetic risk score and smoking phenotypes in TAG and UK Biobank. Age at initiation in log units.

There was evidence that BMI was causally associated with increased likelihood of smoking initiation (Fig. 1; Supplementary Material, Tables S1 and S2). In inverse variance weighted (IVW) MR analysis combining the TAG and UK Biobank results, a one SD increase in BMI increased the odds of being an ever rather than a never smoker by 19% (OR: 1.19, 95% CI: 1.11–1.27). Findings from weighted median, MR Egger and mode weighted regression were consistent with a positive association with smoking initiation, although magnitudes of association were lower in median and weighted mode regression. In MR Egger analysis, there was no clear evidence for directional pleiotropy.

We also found some evidence for a causal effect of higher BMI on smoking heaviness within smokers (Fig. 1). In IVW analysis, each SD increase in BMI increased smoking heaviness by 1.45 (95% CI: 1.03–1.86) additional cigarettes per day. Estimates of these associations were similar for median and weighted mode regression. However, the combined estimate from MR Egger was not consistent with the findings from IVW (β = 0.04, 95% CI: −0.94 to 1.03).

A one SD increase in BMI was associated with a −0.01 log unit decrease in age at initiation (95% CI: −0.02 to 0.0003) in IVW analysis. Results from the other analytical approaches were consistent with this effect but were imprecise (Fig. 1). There was no clear evidence using any of the approaches for a causal effect of BMI on smoking cessation (Fig. 1).

Finally, the analysis of lifetime smoking provided clear evidence in the IVW analysis for a causal effect of BMI on increased lifetime smoking behaviour (β = 0.12, 95% CI: 0.08–0.16; Supplementary Material, Table S2). Weighted median and MR Egger estimates supported the direction of effect but the weighted mode estimate showed no clear evidence of an effect (β = −0.01, 95% CI: −0.07 to 0.06). The MR Egger intercept indicated no clear evidence of directional pleiotropy.

Results were similar for males and females in UK Biobank (*P*-values for heterogeneity in comparisons of IVW analyses all >0.2; Supplementary Material, Tables S3 and S4).

### MR analysis of effect of BMI on DNA methylation

In the ALSPAC mothers, DNA methylation at aryl-hydrocarbon receptor repressor (*AHRR*) was negatively associated with being a smoker and with cigarettes per day (Supplementary Material, Table S5).

There was evidence for a causal effect of BMI on *AHRR* DNA methylation in the ALSPAC mothers in ARIES (Table 1). In IVW MR analysis, a one SD increase in BMI decreased *AHRR* DNA methylation by 0.33 SD (95% CI: −0.55 to −0.11) in samples taken ~18 years post pregnancy and by 0.23 SD (95% CI: −0.47 to 0.01) in the antenatal samples. Evidence from the pleiotropy robust methods were consistent with the results from IVW analysis, but evidence for associations in the antenatal samples was weak using these approaches.

**Table 1:** MR of causal effect of BMI on *AHRR* methylation (cg05575921) in ARIES (N = up to 846)

	**Follow-up methylation (mean age, 47 years)**		**Antenatal methylation (mean age, 29 years)**	
	**Beta (95% CI)**	***P***	**Beta (95% CI)**	***P***
**Inverse variance weighted**	−0.33 (−0.55 to −0.11)	0.004	−0.23 (−0.47 to 0.01)	0.06
**MR Egger slope** **MR Egger intercept**	−0.72 (−1.25 to −0.19) 0.01 (−0.003 to 0.025)	0.008 0.11	−0.33 (−0.92 to 0.25) 0.003 (−0.01 to 0.02)	0.26 0.70
**Weighted median regression**	−0.39 (−0.75 to −0.02)	0.04	−0.12 (−0.51 to 0.26)	0.53
**Weighted mode regression**	−0.54 (−1.00 to −0.08)	0.02	−0.19 (−0.68 to 0.31)	0.46

Using 96 SNPs from Locke *et al*. GWAS. Coefficients represent SD change in methylation per SD change in BMI, adjusted for age, PCs, cell counts and batch.

### MR analysis of effect of BMI on cotinine levels

Using data from the cotinine GWAS, we found no clear evidence for a causal effect of BMI on cotinine levels (beta from IVW: 0.05 SD, 95% CI: −0.13 to 0.23; Table 2).

**Table 2:** MR of causal effect of BMI on cotinine (N = up to 4548)

	**Cotinine (SD)**	
	**Beta (95% CI)**	***P***
**Inverse variance weighted**	0.05 (−0.13 to 0.23)	0.62
**MR Egger slope** **MR Egger intercept**	0.02 (−0.41 to 0.46) 0.001 (−0.01 to 0.01)	0.91 0.92
**Weighted median regression**	0.03 (−0.26 to 0.32)	0.84
**Weighted mode regression**	−0.005 (−0.370 to 0.360)	0.98

Using 95 SNPs from Locke *et al*. GWAS. Coefficients represent SD change in cotinine per SD change in BMI. I-squared for heterogeneity in IVW analysis: 0%, *P* = 0.87.

### MR analysis of effect of BMI on NMR

Across the FinnTwin, FINRISK and Young Finns Study (YFS) studies, there was suggestive evidence that higher BMI was associated with lower NMR (−0.45 per SD increased in BMI, 95% CI: −0.78 to −0.12 in IVW analysis). The magnitude of association was consistent across the other approaches; however, there was a large amount of heterogeneity between the studies for weighted median and weighted mode analyses. Clear evidence for a negative association between BMI and NMR was only seen in the FinnTwin study (Supplementary Material, Table S6).

### MR analysis of the effect of smoking heaviness on BMI

Consistent with previous studies (7,8), the minor allele of the smoking heaviness related variant, rs16969968, increased number of cigarettes smoked per day by 0.95 (95% CI: 0.79–1.11, N = 22 568) and decreased BMI in current (beta per minor allele: −0.21, 95% CI: −0.29 to −0.13, N = 32 685), but not former (beta per minor allele: 0.01, 95% CI: −0.03 to 0.05, N = 116 158) or never smokers (beta per minor allele: 0.02, 95% CI: −0.02 to 0.05, N = 181 333, *P* for interaction between smoking groups<0.001) in UK Biobank.

## Discussion

Using data from multiple cohorts, we find that higher BMI increases the likelihood of becoming a smoker and increases smoking heaviness within current smokers. This finding is supported by the analysis of lifetime smoking and the negative association between the BMI genetic risk score and *AHRR* methylation (which is hypomethylated among smokers). However, the BMI genetic risk score was not associated with cotinine levels and showed some evidence of a negative association with the NMR, which we might expect to reduce cigarette consumption (25). In agreement with previous findings (7), we showed that heavier smoking lowers BMI. Taken together, these results suggest that there may be bidirectional causal effects between smoking phenotypes and BMI, and that these may act in opposing directions. We find no clear evidence for differences between males and females in these effects.

Our results for smoking initiation and cigarettes per day are similar to those presented by Thorgeirsson *et al*. (28), who used the TAG data set but only 32 BMI-related genetic variants, from an earlier GWAS. It is possible that, as they suggest, the effects observed here represent a shared genetic aetiology between BMI and smoking behaviour. However, our results for smoking initiation and cigarettes per day were supported by methods that are more robust to the pleiotropy assumption, MR-Egger and weighted median and weighted mode MR, giving weight to the explanation that this finding represents a causal effect of BMI on smoking uptake and heaviness. This was supported by the negative association we observed between the BMI genetic risk score and DNA methylation at *AHRR*, given that smoking is associated with lower DNA methylation at *AHRR* (39). Our finding could, in part, explain the positive association found between the BMI genetic risk score and certain types of lung cancer (40). Although associations via smoking were ruled out in this analysis, sample sizes for testing associations with smoking behaviour were small.

We did not find clear evidence for an effect of BMI on cotinine levels, which might be expected if having higher BMI increases number of cigarettes smoked per day (and therefore total tobacco intake). It is possible that whilst BMI increases total tobacco intake and therefore absolute cotinine levels, individuals with higher BMI have lower blood cotinine concentration due to higher total blood volume (meaning that cotinine is more diluted in the blood) or greater absorption of cotinine by adipose tissue (23). These opposing effects could lead to a negligible net effect of BMI on cotinine levels.

We observed some evidence for a causal negative effect of BMI on the NMR, although findings should be interpreted with caution as they were very heterogeneous between studies. Although this does not rule out an effect of NMR on BMI mediated through higher tobacco intake, our data provide some support for BMI lowering NMR, the direction hypothesized by Chenoweth *et al*. (27). Given that it is unlikely that BMI affects plasma cotinine and 3′ hydroxycotinine differentially, this could point to an effect of BMI on the enzymes that metabolize these compounds or to indirect effects of BMI via other factors, which may affect NMR (e.g. alcohol consumption, hormone levels) (27). Our findings in relation to NMR demonstrate the potential complexity of the BMI-smoking relationship, with opposing effects on behaviour and metabolism. However, an overall positive effect of BMI on tobacco consumption implies that individuals with higher BMI are still at higher risk of increased tobacco consumption (and therefore the harmful effects of tobacco smoke), even if having higher BMI may reduce levels of metabolites.

**Figure 2:**
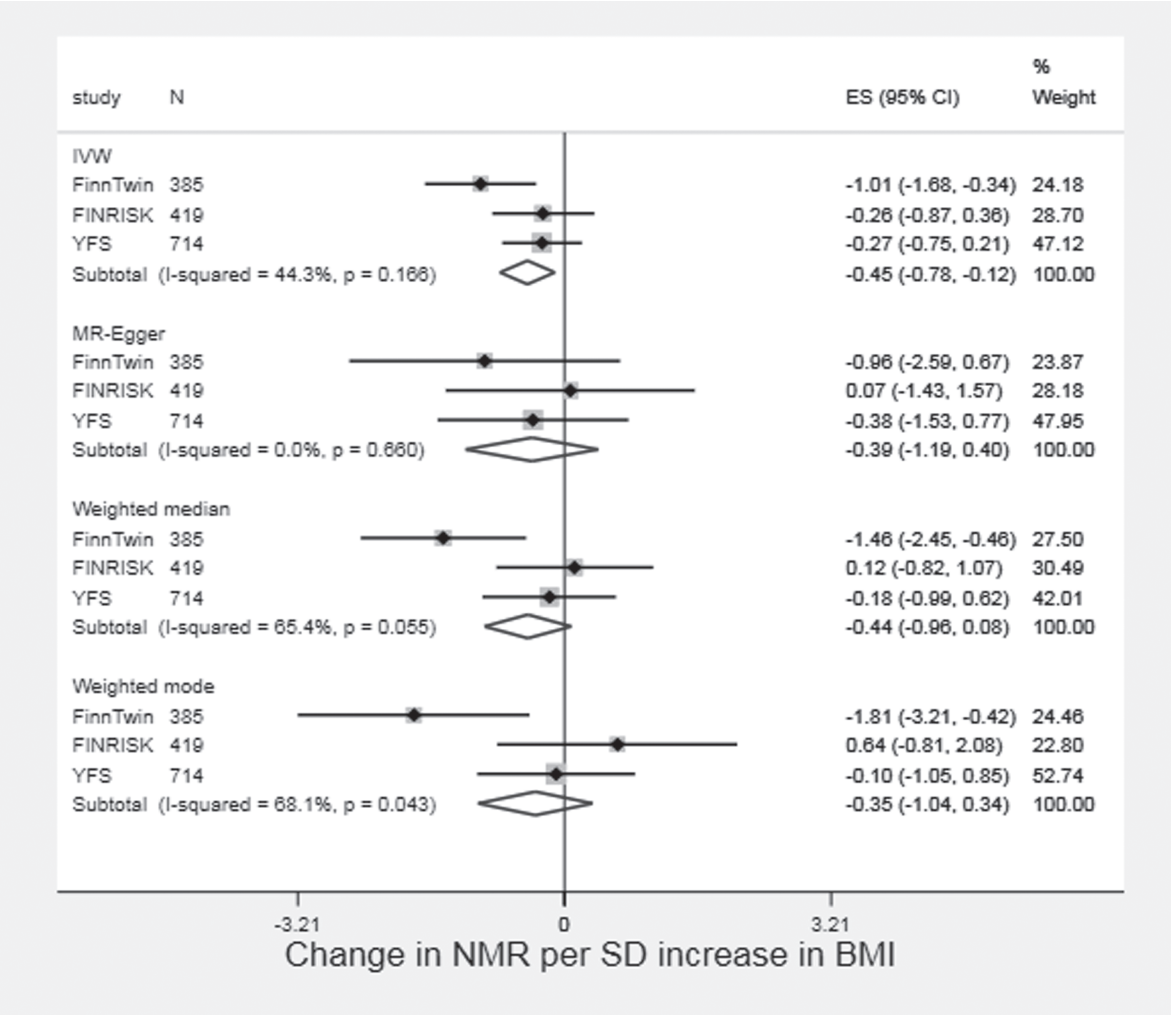
Two sample MR of effect of BMI on NMR in FinnTwin, FINRISK and YFS.

Although we have attempted to explore both behaviour and metabolism in our analyses, it is not clear what the mechanisms underlying the association between higher BMI and smoking initiation and cigarette consumption are. If this is due to individuals with higher BMI having greater concerns about weight control, we might also expect to observe evidence for a causal effect with smoking cessation as fear of weight gain is often provided as a reason for continuing to smoke (10). Importantly, interventions that incorporate weight gain concerns or that aim to tackle weight gain at the same time as smoking cessation may still be effective as weight concerns are not always strongly correlated with or may have non-linear relationships with BMI (19). Given that there is evidence that higher BMI is causally related to lower socioeconomic status, income and educational attainment (41) and that lower educational attainment causes increased smoking (42,43) it is possible that any effect of BMI on smoking could be via these sociodemographic factors.

There are several limitations to this analysis. Firstly, there is sample overlap between the BMI GWAS and the smoking, cotinine, NMR and GWA studies (estimated to be up to 17%), which may have biased the results of our two sample MR analyses in the direction of the observational estimates (44). However, results for smoking behaviour from UK Biobank (which was not included in the BMI GWAS) were highly consistent with those from TAG, suggesting that these results were not driven by bias due to participant overlap. We also repeated the TAG, cotinine and NMR analyses using beta coefficients and standard errors for BMI generated in UK Biobank and these were similar (data not shown). Secondly, we were unable to test associations of the BMI genetic risk score with BMI in the outcome data sets in the two-sample MR analysis. We found some evidence that the association of the BMI genetic risk score with BMI is stronger in current than in former or never smokers in UK Biobank. Therefore, effect sizes should be interpreted with some caution.

In conclusion, our findings support of bidirectional causal effects between BMI and smoking behaviour. Higher BMI leads to increased likelihood of smoking and greater tobacco consumption, but smoking also serves to reduce BMI. Given that BMI and smoking are both major risk factors for disease, this bidirectional causal relationship highlights the need to consider both of these together in prevention strategies. If having higher BMI does increase smoking, interventions aimed at reducing BMI may also help to prevent smoking uptake.

## Materials and Methods

We performed MR analyses using summary data from GWAS and individual level data from the UK Biobank. The sample sizes available to use for our different phenotypes provided 80% power at an alpha level of 5% to detect effect sizes equivalent to OR 1.07 (smoking initiation), OR 1.12 (smoking cessation), 0.06 SD (heaviness of smoking), 0.04 SD (lifetime smoking), 0.3 SD (cotinine levels), 0.5 SD (NMR) and 0.6 SD (methylation). Sample size calculations were performed using http://cnsgenomics.com/shiny/mRnd/ (45).

### Study samples

#### GWAS summary data: BMI

We obtained summary data on the association of genetic variants with BMI from the most recent GIANT BMI GWAS (30). We used the 97 independent single nucleotide polymorphisms (SNPs) identified as reaching genome-wide significance with BMI. Associations between genetic variants and BMI (betas and standard errors) were obtained from the meta-analysis of the European sex-combined data sets (N ≤ 322 135) (30). A full list of SNPs used in each analysis is shown in Supplementary Material, Table S1.

#### GWAS summary data: smoking-related outcomes

We obtained estimates (beta coefficients/odds ratios and standard errors) of the association of BMI-related genetic variants with smoking initiation (ever versus never smoking; N ≤ 74 035), age of initiation (N ≤ 24 114), smoking cessation (former versus current smoking; N ≤ 41 278) and smoking heaviness amongst ever smokers (cigarettes smoked per day;N ≤ 38 101) from the TAG Consortium GWAS (29). We looked up associations of BMI-related SNPs with cotinine in summary data from a published GWAS of cotinine levels in current daily cigarette smokers (N ≤ 4 548) (35) and with the NMR in summary data from a GWAS in cotinine-verified current smokers (36). Summary statistics for the NMR GWAS not adjusted for BMI were obtained from the study authors separately for the Finnish Twin Study (FinnTwin), the YFS and the National FINRISK study.

#### GWAS summary data: DNA methylation

We performed genome-wide association analysis of DNA methylation at the *AHRR* methylation site cg05575921 (the strongest smoking-associated methylation locus identified to date) (39) in the ALSPAC ARIES resource (46). ALSPAC is a longitudinal birth cohort, which recruited 14 541 pregnant women with due dates between 1 April 1991 and 31 December 1992. Information on these women and their children has been collected at clinics and via questionnaires ever since (38,47). Please note that the study website contains details of all the data that is available through a fully searchable data dictionary (http://www.bris.ac.uk/alspac/researchers/data-access/data-dictionary/). Ethics approval for the study was obtained from the ALSPAC Ethics and Law Committee and the Local Research Ethics Committees. The ARIES resource includes 1018 mother offspring pairs. DNA methylation in the mothers was assessed from blood samples taken at two time points: during pregnancy and~18 years later. Genome-wide DNA methylation profiling in ARIES was performed using the Illumina Infinium HumanMethylation450 BeadChip (450 K) array (Illumina, Inc. San Diego, CA) (46). Full details of the GWAS methods are provided in supplementary material. The sample used in the GWAS (N ≤ 846) included smokers and non-smokers. Beta coefficients and standard errors of the association with methylation for each of the BMI-related SNPs were obtained from the GWAS summary statistics.

#### UK Biobank

We also used data on individuals from the UK Biobank, which recruited over 500 000 individuals (aged between 40 and 70 years) in the UK (48). Individuals attended assessment centres between 2006 and 2010, where they completed a questionnaire on lifestyle factors and had blood samples and measurements taken. Individuals were classified as ever smokers if they had smoked more than 100 cigarettes in their lifetime and current smokers if they indicated that they were still smoking. Cigarettes smoked per day amongst current smokers and past regular smokers was reported on a continuous scale. BMI was calculated as weight/height [(kg)/(m)^2^]. Lifetime smoking was calculated from self-reported smoking duration, cessation and heaviness. A full description of score construction is provided elsewhere (49). In this analysis, we included unrelated individuals of white British ancestry (N = 335 921; see supplementary material for details) (50).

### Statistical analysis

In two-sample MR analysis, we calculated the ratio of the SNP-outcome and SNP-exposure associations (the Wald estimator) for each of the 97 BMI-related SNPs (Supplementary Material, Table S1), to give an estimate of the effect of BMI on the outcome. Where BMI-related SNPs were not available in the outcome GWAS, proxy SNPs (with an R-squared value of >0.9 with the original SNP) were used if available. The single SNP estimates were combined in an IVW random effects meta-analysis, as outlined by Burgess and colleagues (51), using the mrrobust package in Stata (52). For the analysis of smoking initiation, we excluded the genetic variant in *BDNF*, as this locus is likely to be pleiotropic and is associated with smoking initiation at genome-wide significance level (29).

Within UK Biobank, we generated a weighted BMI genetic risk score from dosage scores of the 97 SNPs, using the weights from the combined ancestries GIANT analysis (30) and tested the association of the standardized risk score against measured BMI using linear regression. We generated our own outcome summary statistics by calculating associations of each SNP with smoking behaviour phenotypes using logistic or linear regression, adjusted for 10 principal genetic components, and produced causal estimates using the same two-sample MR IVW method as outlined above. We performed primary analyses in the full sample, but also stratified by sex, given evidence from previous literature that the relationship between weight concern and smoking might be stronger in females. Results from TAG and UK Biobank were meta-analysed using inverse variance weighted fixed effects meta-analysis (except for lifetime smoking, where a comparable phenotype was not available in the TAG data).

We also performed analyses that are more robust to potential pleiotropy, MR Egger (32), weighted median regression (33) and the mode-based estimator (34). The MR Egger method is similar to IVW, but allows the intercept of the regression line to change. The intercept is a test of directional pleiotropy; if the intercept differs from zero, this indicates that there is directional pleiotropy. The slope obtained from MR Egger is an estimate of the causal effect after taking into account this directional pleiotropy (32). Weighted median regression generates a consistent estimate of a causal effect even when up to 50% of SNPs are invalid instruments (33). The mode-based estimator method assumes that the most commonly occurring causal effect estimate is a consistent estimate of the true causal effect (34).

In addition, we attempted to replicate previous analyses investigating the causal effect of smoking on BMI (7), using the rs16969968 functional variant in the *CHRNA3-A5-B4* gene cluster, which increases smoking heaviness (cigarettes smoked per day) amongst smokers (53). We regressed the rs16969968 SNP on BMI in never, former and current smokers, adjusting for age, sex and principal components in UK Biobank.

All analyses were conducted in Stata (version 14.1), with the exception of the analysis of lifetime smoking, which was conducted in R (R Core Team, 2013). Scatter plot of the genetic associations with BMI against the genetic associations with major outcome variables are presented in Supplementary Material, Figures S1 and S2.

## Supplementary Material

HMG-2018-EZ-00418-Supplementary-Material_ddy434

## References

[1] World Health Organisation (2009) *Global Health Risks: Mortality and Burden of Disease Attributable to Selected Major Risks*. WHO Press, Geneva.

[2] Audrain-McGovern J. and Benowitz N.L. (2011) Cigarette smoking, nicotine, and body weight. Clin. Pharmacol. Ther., 90, 164–168. 21633341 10.1038/clpt.2011.105 PMC3195407

[3] Dare S., Mackay D.F. and Pell J.P. (2015) Relationship between smoking and obesity: a cross-sectional study of 499,504 middle-aged adults in the UK general population. PLoS One, 10, e0123579. 10.1371/journal.pone.0123579 PMC4401671 25886648

[4] Ma J., Betts N.M. and Hampl J.S. (2000) Clustering of lifestyle behaviors: the relationship between cigarette smoking, alcohol consumption, and dietary intake. Am. J. Health Promot., 15, 107–117. 11194694 10.4278/0890-1171-15.2.107

[5] Healton C.G., Vallone D., McCausland K.L., Xiao H. and Green M.P. (2006) Smoking, obesity, and their co-occurrence in the United States: cross sectional analysis. BMJ, 333, 25–26. 16698804 10.1136/bmj.38840.608704.80 PMC1488756

[6] Davey Smith G. and Ebrahim S. (2003) `Mendelian randomization': can genetic epidemiology contribute to understanding environmental determinants of disease? Int. J. Epidemiol., 32,–22. 10.1093/ije/dyg070 12689998

[7] Freathy R.M., Kazeem G.R., Morris R.W., Johnson P.C., Paternoster L., Ebrahim S., Hattersley A.T., Hill A., Hingorani A.D., Holst C. et al. (2011) Genetic variation at CHRNA5-CHRNA3-CHRNB4 interacts with smoking status to influence body mass index. Int. J. Epidemiol., 40, 1617–1628. 21593077 10.1093/ije/dyr077 PMC3235017

[8] Asvold B.O., Bjorngaard J.H., Carslake D., Gabrielsen M.E., Skorpen F., Davey Smith G. and Romundstad P.R. (2014) Causal associations of tobacco smoking with cardiovascular risk factors: a Mendelian randomization analysis of the HUNT study in Norway. Int. J. Epidemiol., 43, 1458–1470. 24867305 10.1093/ije/dyu113

[9] Morris R.W., Taylor A.E., Fluharty M.E., Bjorngaard J.H., Asvold B.O., Elvestad Gabrielsen M., Campbell A., Marioni R., Kumari M., Korhonen T. et al. (2015) Heavier smoking may lead to a relative increase in waist circumference: evidence for a causal relationship from a Mendelian randomisation meta-analysis. BMJ Open, 5, e008808. 10.1136/bmjopen-2015-008808 PMC4538266 26264275

[10] Farley A.C., Hajek P., Lycett D. and Aveyard P. (2012) Interventions for preventing weight gain after smoking cessation. Cochrane Database Syst. Rev., 1, CD006219. 10.1002/14651858.CD006219.pub3 22258966

[11] Jain P., Danaei G., Robins J.M., Manson J.E. and Hernan M.A. (2016) Smoking cessation and long-term weight gain in the Framingham Heart Study: an application of the parametric g-formula for a continuous outcome. Eur. J. Epidemiol., 31, 1223–1229. 27704230 10.1007/s10654-016-0200-4 PMC5759957

[12] Filozof C., Fernandez Pinilla M.C. and Fernandez-Cruz A. (2004) Smoking cessation and weight gain. Obesity, 5, 95–103. 10.1111/j.1467-789X.2004.00131.x 15086863

[13] Aveyard P., Lycett D. and Farley A. (2012) Managing smoking cessation-related weight gain. Pol. Arch. Intern. Med., 122, 494–498. 23123526

[14] Yang M., Chen H., Johnson M.L., Essien E.J., Peters R.J., Wang X. and Abughosh S. (2016) Comparative effectiveness of smoking cessation medications to attenuate weight gain following cessation. Subst. Use Misuse, 51, 586–597. 27007975 10.3109/10826084.2015.1126744

[15] Davey Smith G., Bracha Y., Svendsen K.H., Neaton J.D., Haffner S.M., Kuller L.H. and Intervention M.R.F. (2005) Incidence of type 2 diabetes in the randomized multiple risk factor intervention trial. Ann. Intern. Med., 142, 313–322. 15738450 10.7326/0003-4819-142-5-200503010-00006

[16] Clark M.M., Decker P.A., Offord K.P., Patten C.A., Vickers K.S., Croghan I.T., Hays J.T., Hurt R.D. and Dale L.C. (2004) Weight concerns among male smokers. Addict. Behav., 29, 1637–1641. 15451131 10.1016/j.addbeh.2004.02.034

[17] Winter A.L., Guia N.A. de, Ferrence R. and Cohen J.E. (2002) The relationship between body weight perceptions, weight control behaviours and smoking status among adolescents. Can. J. Public Health, 93, 362–365. 12353458 10.1007/BF03404570 PMC6979969

[18] Tomeo C.A., Field A.E., Berkey C.S., Colditz G.A. and Frazier A.L. (1999) Weight concerns, weight control behaviors, and smoking initiation. Pediatrics, 104, 918–924. 10506235 10.1542/peds.104.4.918

[19] Calzo J.P., Sonneville K.R., Haines J., Blood E.A., Field A.E. and Austin S.B. (2012) The development of associations among body mass index, body dissatisfaction, and weight and shape concern in adolescent boys and girls. J. Adolesc. Health, 51, 517–523. 23084175 10.1016/j.jadohealth.2012.02.021 PMC3479441

[20] Howe L.J., Trela-Larsen L., Taylor M., Heron J., Munafo M.R. and Taylor A.E. (2017) Body mass index, body dissatisfaction and adolescent smoking initiation. Drug Alcohol Depend., 178, 143–149. 28647682 10.1016/j.drugalcdep.2017.04.008 PMC5558147

[21] Strine T.W., Mokdad A.H., Dube S.R., Balluz L.S., Gonzalez O., Berry J.T., Manderscheid R. and Kroenke K. (2008) The association of depression and anxiety with obesity and unhealthy behaviors among community-dwelling US adults. Gen. Hosp. Psychiatry, 30, 127–137. 18291294 10.1016/j.genhosppsych.2007.12.008

[22] Broek N. van den, Treur J.L., Larsen J.K., Verhagen M., Verweij K.J.H. and Vink J. (2018) Causal associations between body mass index and mental health: a Mendelian randomization study. J. Epidemiol. Community Health*,* 72*,* 708–710. 29666151 10.1136/jech-2017-210000

[23] Jain R.B. and Bernert J.T. (2010) Effect of body mass index and total blood volume on serum cotinine levels among cigarette smokers: NHANES 1999-2008. Clin. Chim. Acta, 411, 1063–1068. 20361952 10.1016/j.cca.2010.03.040

[24] Prather R.D., Tu T.G., Rolf C.N. and Gorsline J. (1993) Nicotine pharmacokinetics of Nicoderm (nicotine transdermal system) in women and obese men compared with normal-sized men. J. Clin. Pharmacol., 33, 644–649. 8366189 10.1002/j.1552-4604.1993.tb04718.x

[25] Chenoweth M.J., Schnoll R.A., Novalen M., Hawk L.W. Jr, George T.P., Cinciripini P.M., Lerman C. and Tyndale R.F. (2016) The nicotine metabolite ratio is associated with early smoking abstinence even after controlling for factors that influence the nicotine metabolite ratio. Nicotine Tob. Res., 18, 491–495. 26069034 10.1093/ntr/ntv125 PMC6220835

[26] Chenoweth M.J., O'Loughlin J., Sylvestre M.P. and Tyndale R.F. (2013) CYP2A6 slow nicotine metabolism is associated with increased quitting by adolescent smokers. Pharmacogenet. Genomics, 23, 232–235. 23462429 10.1097/FPC.0b013e32835f834d PMC3744214

[27] Chenoweth M.J., Novalen M., Hawk L.W. Jr, Schnoll R.A., George T.P., Cinciripini P.M., Lerman C. and Tyndale R.F. (2014) Known and novel sources of variability in the nicotine metabolite ratio in a large sample of treatment-seeking smokers. Cancer Epidemiol. Biomarkers Prev., 23, 1773–1782. 25012994 10.1158/1055-9965.EPI-14-0427 PMC4154993

[28] Thorgeirsson T.E., Gudbjartsson D.F., Sulem P., Besenbacher S., Styrkarsdottir U., Thorleifsson G., Walters G.B., TAG Consortium, Oxford-GSK Consortium, ENGAGE Consortium et al. (2013) A common biological basis of obesity and nicotine addiction. Transl. Psychiatry, 3, e308. 24084939 10.1038/tp.2013.81 PMC3818010

[29] Furberg H., Kim Y., Dackor J., Boerwinkle E., Franceschini N., Ardissino D., Bernardinelli L., Mannucci P.M., Mauri F., Merlini P.A. et al. (2010) Genome-wide meta-analyses identify multiple loci associated with smoking behavior. Nat. Genet., 42, 441–447. 20418890 10.1038/ng.571 PMC2914600

[30] Locke A.E., Kahali B., Berndt S.I., Justice A.E., Pers T.H., Day F.R., Powell C., Vedantam S., Buchkovich M.L., Yang J. et al. (2015) Genetic studies of body mass index yield new insights for obesity biology. Nature, 518, 197–206. 25673413 10.1038/nature14177 PMC4382211

[31] Carreras-Torres R., Johansson M., Haycock P.C., Relton C.L., Davey Smith G., Brennan P. and Martin R.M. (2018) Role of obesity in smoking behaviour: Mendelian randomisation study in UK Biobank. BMJ, 361, k1767. 10.1136/bmj.k1767 PMC5953237 29769355

[32] Bowden J., Davey Smith G. and Burgess S. (2015) Mendelian randomization with invalid instruments: effect estimation and bias detection through Egger regression. Int. J. Epidemiol., 44, 512–525. 26050253 10.1093/ije/dyv080 PMC4469799

[33] Bowden J., Davey Smith G., Haycock P.C. and Burgess S. (2016) Consistent estimation in Mendelian randomization with some invalid instruments using a weighted median estimator. Genet. Epidemiol., 40, 304–314. 27061298 10.1002/gepi.21965 PMC4849733

[34] Hartwig F.P., Davey Smith G. and Bowden J. (2017) Robust inference in summary data Mendelian randomization via the zero modal pleiotropy assumption. Int. J. Epidemiol., 46, 1985–1998. 29040600 10.1093/ije/dyx102 PMC5837715

[35] Ware J.J., Chen X., Vink J., Loukola A., Minica C., Pool R., Milaneschi Y., Mangino M., Menni C., Chen J. et al. (2016) Genome-wide meta-analysis of cotinine levels in cigarette smokers identifies locus at 4q13.2. Sci. Rep., 6, 20092. 10.1038/srep20092 PMC4735517 26833182

[36] Loukola A., Buchwald J., Gupta R., Palviainen T., Hallfors J., Tikkanen E., Korhonen T., Ollikainen M., Sarin A.P., Ripatti S. et al. (2015) A genome-wide association study of a biomarker of nicotine metabolism. PLoS Genet., 11, e1005498. 10.1371/journal.pgen.1005498 PMC4583245 26407342

[37] Allen N.E., Sudlow C., Peakman T., Collins R. and U.K Biobank (2014) UK Biobank data: come and get it. *Sci. Transl. Med.*, 6, 224ed224. 10.1126/scitranslmed.3008601 24553384

[38] Fraser A., Macdonald-Wallis C., Tilling K., Boyd A., Golding J., Davey Smith G., Henderson J., Macleod J., Molloy L., Ness A. et al. (2013) Cohort profile: the Avon Longitudinal Study of Parents and Children: ALSPAC mothers cohort. Int. J. Epidemiol., 42, 97–110. 22507742 10.1093/ije/dys066 PMC3600619

[39] Zeilinger S., Kuhnel B., Klopp N., Baurecht H., Kleinschmidt A., Gieger C., Weidinger S., Lattka E., Adamski J., Peters A. et al. (2013) Tobacco smoking leads to extensive genome-wide changes in DNA methylation. PLoS One, 8: e63812. 23691101 10.1371/journal.pone.0063812 PMC3656907

[40] Carreras-Torres R., Haycock P.C., Relton C.L., Martin R.M., Davey Smith G., Kraft P., Gao C., Tworoger S., Le Marchand L., Wilkens L.R. et al. (2016) The causal relevance of body mass index in different histological types of lung cancer: a Mendelian randomization study. Sci. Rep., 6, 31121. 10.1038/srep31121 PMC4973233 27487993

[41] Tyrrell J., Jones S.E., Beaumont R., Astley C., Lovell R., Yaghootkar H., Tuke M., Ruth K.S., Freathy R.M., Hirschhorn J.N. et al. (2016) Higher BMI leads to lower socioeconomic status: a Mendelian randomisation study in the UK Biobank. Diabetes, 65, A431–A431. 10.1136/bmj.i582 PMC4783516 26956984

[42] Gage S.H., Bowden J., Davey Smith G. and Munafo M. (2018) Investigating causality in associations between education and smoking: a two-sample Mendelian randomization study. Int. J. Epidemiol., 47, 1131–1140. 29961807 10.1093/ije/dyy131 PMC6124626

[43] Tillmann T., Vaucher J., Okbay A., Pikhart H., Peasey A., Kubinova R., Pajak A., Tamosiunas A., Malyutina S., Hartwig F.P. et al. (2017) Education and coronary heart disease: mendelian randomisation study. BMJ, 358, j3542. 10.1136/bmj.j3542 PMC5594424 28855160

[44] Burgess S., Davies N.M. and Thompson S.G. (2016) Bias due to participant overlap in two-sample Mendelian randomization. Genet. Epidemiol., 40, 597–608. 27625185 10.1002/gepi.21998 PMC5082560

[45] Brion M.-J.A., Shakhbazov K. and Visscher P.M. (2013) Calculating statistical power in Mendelian randomization studies. Int. J. Epidemiol., 42, 1497–1501. 24159078 10.1093/ije/dyt179 PMC3807619

[46] Relton C.L., Gaunt T., McArdle W., Ho K., Duggirala A., Shihab H., Woodward G., Lyttleton O., Evans D.M., Reik W. et al. (2015) Data Resource Profile: Accessible Resource for Integrated Epigenomic Studies (ARIES). Int. J. Epidemiol., 44, 1181–1190. 25991711 10.1093/ije/dyv072 PMC5593097

[47] Boyd A., Golding J., Macleod J., Lawlor D.A., Fraser A., Henderson J., Molloy L., Ness A., Ring S. and Davey Smith G. (2013) Cohort profile: the 'Children of the 90s'—the index offspring of the Avon Longitudinal Study of Parents and Children. Int. J. Epidemiol., 42, 111–127. 22507743 10.1093/ije/dys064 PMC3600618

[48] Collins R. (2012) What makes UK Biobank special? Lancet, 379, 1173–1174. 22463865 10.1016/S0140-6736(12)60404-8

[49] Wootton R.E., Richmond R.C., Suitfizand B.G., Lawn R.B., Sallis H.M., Taylor G.M.J., Jones H.J., Zammit S., Davey Smith G. and Munafo M.R. (2018) Causal effects of lifetime smoking on risk for depression and schizophrenia: evidence from a Mendelian randomisation study. *bioRxiv* doi:10.1101/381301. PMC7610182 31689377

[50] Mitchell R., Hemani G., Dudding T. and Paternoster L. (2017) UK Biobank Genetic Data: MRC-IEU Quality Control. Version 1, 10.5523/bris.3074krb6t2frj29yh2b03x3wxj.

[51] Burgess S., Scott R.A., Timpson N.J., Davey Smith G., Thompson S.G. and Consortium E.-I. (2015) Using published data in Mendelian randomization: a blueprint for efficient identification of causal risk factors. Eur. J. Epidemiol., 30, 543–552. 25773750 10.1007/s10654-015-0011-z PMC4516908

[52] Spiller W., Davies N.M. and Palmer T.M. (2017) Software application profile: mrrobust—a tool for performing two-sample summary Mendelian randomization analyses. Int. J. Epidemiol. doi:10.1093/ije/dyy195.

[53] Ware J.J., Bree M.B. van den and Munafo M.R. (2011) Association of the CHRNA5-A3-B4 gene cluster with heaviness of smoking: a meta-analysis. Nicotine Tob. Res., 13, 1167–1175. 22071378 10.1093/ntr/ntr118 PMC3223575

