# Supplementary material for: The effect of body mass index on smoking behaviour and nicotine metabolism: a Mendelian randomization study
Source: Hum Mol Genet. 2018 Dec 18;28(8):1322–30. doi: 10.1093/hmg/ddy434 (PMC6452214; doi:10.1093/hmg/ddy434)
Supplement: HMG-2018-EZ-00418-Supplementary-Material_ddy434 [file hmg-2018-ez-00418-supplementary-material_ddy434.docx]

**Supplementary Material**

**ARIES**

ARIES mothers were genotyped using the Illumina Human660W-quad genome-wide SNP genotyping platform (Illumina Inc., San Diego, CA, USA) at the Centre National de Génotypage (CNG; Paris, France). Individuals were excluded based on non-European ancestry, missingness, relatedness, gender mismatches and heterozygosity. PLINK (v1.07) (1) was used to carry out quality control measures on an initial set of 10,015 subjects (including non-ARIES ALSPAC participants) and 557,124 directly genotyped SNPs. Following QC, the final directly genotyped dataset contained 526,688 SNP loci.

Imputation was performed to increase the SNP density for all genotyped mothers and children combined. Genotypes were phased together using ShapeIt (version 2, revision 727) and then imputed against the 1000 Genomes reference panel (phase 1, version 3, phased using ShapeIt version 2, December 2013, using all populations) using Impute (v2.2.2). Genotypes were filtered to have Hardy–Weinberg equilibrium p > 5 × 10−7, MAF >1 % and imputation info score >0.8. Best guess genotypes were used for subsequent analysis. The final imputed dataset used for the analyses presented here contained 8,074,398 loci.

Methylation data were normalised in R with the wateRmelon package (2) using the Touleimat and Tost (3) algorithm to reduce the non-biological differences between probes. As was done previously (4), *AHRR* methylation (cg05575921) was rank-normalised to remove outliers and regressed on the following covariates: age, the top ten ancestry principal components, bisulphite conversion batch and estimated white blood cell counts (using an algorithm based on differential methylation between cell types (5). Residuals were then taken forward and SNP effects were obtained in PLINK1.07 using exact linear regression.

**UK Biobank**

UK Biobank is a population-based health research resource consisting of approximately 500,000 people, aged between 38 years and 73 years, who were recruited between the years 2006 and 2010 from across the UK 1. Particularly focused on identifying determinants of human diseases in middle-aged and older individuals, participants provided a range of information (such as demographics, health status, lifestyle measures, cognitive testing, personality self-report, and physical and mental health measures) via questionnaires and interviews; anthropometric measures, BP readings and samples of blood, urine and saliva were also taken (data available at www.ukbiobank.ac.uk). A full description of the study design, participants and quality control (QC) methods have been described in detail previously2. UK Biobank received ethical approval from the Research Ethics Committee (REC reference for UK Biobank is 11/NW/0382).

**Genotyping methods**

**Genotyping and imputation**

The full data release contains the cohort of successfully genotyped samples (n=488,377). 49,979 individuals were genotyped using the UK BiLEVE array and 438,398 using the UK Biobank axiom array. Pre-imputation QC, phasing and imputation are described elsewhere (6). In brief, prior to phasing, multiallelic SNPs or those with MAF ≤1% were removed. Phasing of genotype data was performed using a modified version of the SHAPEIT2 algorithm (7). Genotype imputation to a reference set combining the UK10K haplotype and HRC reference panels (8) was performed using IMPUTE2 algorithms (9) . The analyses presented here were restricted to autosomal variants within the HRC site list using a graded filtering with varying imputation quality for different allele frequency ranges. Therefore, rarer genetic variants are required to have a higher imputation INFO score (Info>0.3 for MAF >3%; Info>0.6 for MAF 1-3%; Info>0.8 for MAF 0.5-1%; Info>0.9 for MAF 0.1- 0.5%) with MAF and Info scores having been recalculated on an in house derived ‘European’ subset. Both rs16969968 and rs3025343 were imputed with an info score of 1 and did not show clear evidence of deviation from hardy Weinberg equilibrium (p>0.6).

**Data quality control**

Individuals with sex-mismatch (derived by comparing genetic sex and reported sex) or individuals with sex-chromosome aneuploidy were excluded from the analysis (n=814).

**Ancestry**

We restricted the sample to individuals of white British ancestry who self-report as “White British” and who have very similar ancestral backgrounds according to the PCA (n=409,703), as described by Bycroft (6).

**Degree of relatedness**

Estimated kinship coefficients using the KING toolset (10) identified 107,162 pairs of individuals (6). An in-house algorithm was then applied to this list and preferentially removed the individuals related to the greatest number of other individuals until no related pairs remain. These individuals were excluded (n=79,448). Additionally, 2 individuals were removed due to them relating to a very large number (>200) of individuals.

**Table S1. Genetic variants used in the analyses**

| **Genetic variant** | **Beta** | **Effect allele** | **TAG GWAS** | **UK Biobank** | **Cotinine GWAS** | **ALSPAC** | **FINRISK** | **FINNTWIN** | **YFS** |
| --- | --- | --- | --- | --- | --- | --- | --- | --- | --- |
| rs11165643 | .022 | T | Yes | Yes | Yes | Yes | Yes | Yes | Yes |
| rs11583200 | .017 | C | Yes | Yes | Yes | Yes | Yes | Yes | rs6700902 |
| rs12401738 | .02 | A | Yes | Yes | Yes | Yes | Yes | Yes | Yes |
| rs12566985 | .024 | G | Yes | Yes | Yes | Yes | Yes | Yes | Yes |
| rs17024393 | .061 | C | Yes | Yes | Yes | Yes | Yes | Yes | Yes |
| rs2820292 | .018 | C | Yes | Yes | Yes | Yes | Yes | Yes | Yes |
| rs3101336 | .032 | C | Yes | Yes | Yes | Yes | Yes | Yes | rs1460942 |
| rs543874 | .05 | G | Yes | Yes | Yes | Yes | Yes | Yes | Yes |
| rs657452 | .023 | A | Yes | Yes | Yes | Yes | Yes | Yes | rs1343425 |
| rs977747 | .017 | T | Yes | Yes | Yes | Yes | Yes | Yes | Yes |
| rs1016287 | .023 | T | Yes | Yes | Yes | Yes | Yes | Yes | Yes |
| rs10182181 | .031 | G | Yes | Yes | Yes | Yes | Yes | Yes | rs59086897 |
| rs11126666 | .02 | A | Yes | Yes | Yes | Yes | Yes | Yes | Yes |
| rs11688816 | .015 | G | Yes | Yes | Yes | Yes | Yes | Yes | rs6714241 |
| rs13021737 | .06 | G | Yes | Yes | Yes | Yes | Yes | Yes | Yes |
| rs1460676 | .021 | C | Yes | Yes | Yes | Yes | Yes | Yes | Yes |
| rs1528435 | .018 | T | Yes | Yes | Yes | Yes | Yes | Yes | Yes |
| rs17203016 | .021 | G | Yes | Yes | Yes | Yes | Yes | Yes | N/A |
| rs2121279 | .024 | T | Yes | Yes | Yes | Yes | Yes | Yes | Yes |
| rs2176040 | .015 | A | Yes | Yes | Yes | Yes | Yes | Yes | Yes |
| rs492400 | .015 | C | Yes | Yes | Yes | Yes | Yes | Yes | Yes |
| rs7599312 | .021 | G | Yes | Yes | Yes | Yes | Yes | Yes | rs6735267 |
| rs13078960 | .029 | G | Yes | Yes | Yes | Yes | Yes | Yes | Yes |
| rs1516725 | .045 | C | Yes | Yes | Yes | Yes | Yes | Yes | Yes |
| rs16851483 | .048 | T | Yes | Yes | Yes | Yes | Yes | Yes | Yes |
| rs2365389 | .02 | C | Yes | Yes | Yes | Yes | Yes | Yes | Yes |
| rs3849570 | .018 | A | Yes | Yes | Yes | Yes | Yes | Yes | Yes |
| rs6804842 | .018 | G | Yes | Yes | Yes | Yes | Yes | Yes | N/A* |
| rs10938397 | .04 | G | Yes | Yes | Yes | Yes | Yes | Yes | Yes |
| rs11727676 | .037 | T | Yes | Yes | Yes | Yes | Yes | Yes | Yes |
| rs13107325 | .047 | T | Yes | Yes | Yes | Yes | rs13135092 | Yes | rs13135092 |
| rs17001654 | .03 | G | Yes | Yes | Yes | Yes | Yes | Yes | Yes |
| rs2112347 | .025 | T | Yes | Yes | Yes | Yes | Yes | Yes | Yes |
| rs7715256 | .017 | G | Yes | Yes | Yes | Yes | Yes | Yes | Yes |
| rs13191362 | .029 | A | Yes | Yes | Yes | Yes | Yes | Yes | Yes |
| rs13201877 | .024 | G | Yes | Yes | Yes | Yes | Yes | Yes | Yes |
| rs2033529 | .018 | G | Yes | rs3734555 | Yes | Yes | Yes | Yes | N/A |
| rs205262 | .021 | G | Yes | Yes | Yes | Yes | Yes | Yes | rs6457796 |
| rs2207139 | .045 | G | Yes | Yes | Yes | Yes | Yes | Yes | Yes |
| rs9374842 | .02 | T | Yes | Yes | Yes | Yes | Yes | Yes | Yes |
| rs9400239 | .017 | C | Yes | Yes | Yes | Yes | Yes | Yes | rs10457180 |
| rs1167827 | .02 | G | Yes | Yes | Yes | Yes | Yes | Yes | N/A* |
| rs2245368 | .029 | C | Yes | Yes | N/A | N/A | Yes | Yes | Yes |
| rs6465468 | .016 | T | Yes | Yes | Yes | N/A | Yes | Yes | Yes |
| rs9641123 | .019 | C | Yes | Yes | Yes | Yes | Yes | Yes | Yes |
| rs16907751 | .033 | C | Yes | Yes | Yes | Yes | Yes | Yes | Yes |
| rs17405819 | .022 | T | Yes | Yes | Yes | Yes | Yes | Yes | Yes |
| rs2033732 | .018 | C | Yes | Yes | Yes | Yes | Yes | Yes | Yes |
| rs10733682 | .019 | A | Yes | Yes | Yes | Yes | Yes | Yes | N/A |
| rs10968576 | .025 | G | Yes | Yes | Yes | Yes | Yes | Yes | rs17770163 |
| rs1928295 | .018 | T | Yes | Yes | Yes | Yes | Yes | Yes | N/A* |
| rs4740619 | .017 | T | Yes | Yes | Yes | Yes | Yes | Yes | N/A* |
| rs6477694 | .017 | C | Yes | Yes | Yes | Yes | Yes | Yes | rs6477694 |
| rs11191560 | .031 | C | Yes | Yes | Yes | Yes | Yes | Yes | Yes |
| rs17094222 | .025 | C | Yes | Yes | Yes | Yes | Yes | Yes | Yes |
| rs7899106 | .038 | G | Yes | Yes | Yes | Yes | Yes | Yes | Yes |
| rs7903146 | .024 | C | Yes | Yes | Yes | Yes | Yes | Yes | rs35198068 |
| rs11030104 | .042 | A | Yes | Yes | Yes | Yes | Yes | Yes | rs4923464 |
| rs12286929 | .021 | G | Yes | Yes | Yes | Yes | Yes | Yes | Yes |
| rs2176598 | .019 | T | Yes | Yes | Yes | Yes | Yes | Yes | Yes |
| rs3817334 | .026 | T | Yes | Yes | Yes | Yes | Yes | Yes | Yes |
| rs4256980 | .021 | G | Yes | Yes | Yes | Yes | Yes | Yes | Yes |
| rs11057405 | .03 | G | Yes | Yes | N/A | Yes | Yes | Yes | N/A |
| rs7138803 | .032 | A | Yes | Yes | Yes | Yes | Yes | Yes | rs7306275 |
| rs12016871 | .03 | T | Yes | rs9581854 | rs9581855 | rs9581854 | rs9581854 | rs9581854 | rs9581854 |
| rs12429545 | .032 | A | Yes | Yes | Yes | Yes | Yes | Yes | rs9568867 |
| rs1441264 | .017 | A | Yes | Yes | Yes | Yes | Yes | Yes | rs1576655 |
| rs9540493 | .018 | A | Yes | Yes | Yes | Yes | Yes | Yes | Yes |
| rs10132280 | .022 | C | Yes | Yes | Yes | Yes | Yes | Yes | Yes |
| rs11847697 | .037 | T | Yes | Yes | Yes | Yes | Yes | Yes | Yes |
| rs12885454 | .02 | C | Yes | Yes | Yes | Yes | Yes | Yes | Yes |
| rs7141420 | .023 | T | Yes | Yes | Yes | Yes | Yes | Yes | N/A |
| rs16951275 | .03 | T | Yes | Yes | Yes | Yes | Yes | Yes | Yes |
| rs3736485 | .016 | A | Yes | Yes | Yes | Yes | Yes | Yes | Yes |
| rs7164727 | .019 | T | Yes | Yes | Yes | Yes | Yes | Yes | rs2162269 |
| rs12446632 | .04 | G | Yes | Yes | Yes | Yes | Yes | Yes | rs72771045 |
| rs1558902 | .081 | A | Yes | Yes | Yes | Yes | Yes | Yes | Yes |
| rs2080454 | .017 | C | Yes | Yes | Yes | Yes | Yes | Yes | Yes |
| rs2650492 | .021 | A | Yes | Yes | Yes | Yes | Yes | Yes | rs2726034 |
| rs3888190 | .031 | A | Yes | Yes | Yes | Yes | Yes | Yes | Yes |
| rs4787491 | .015 | G | Yes | Yes | Yes | Yes | Yes | Yes | Yes |
| rs758747 | .023 | T | Yes | Yes | Yes | Yes | Yes | Yes | N/A |
| rs9925964 | .02 | A | Yes | Yes | Yes | Yes | Yes | Yes | Yes |
| rs1000940 | .018 | G | Yes | Yes | Yes | Yes | Yes | Yes | rs10792 |
| rs12940622 | .018 | G | Yes | Yes | Yes | Yes | Yes | Yes | rs9910745 |
| rs9914578 | .02 | G | Yes | Yes | Yes | Yes | Yes | Yes | Yes |
| rs1808579 | .016 | C | Yes | Yes | Yes | Yes | Yes | Yes | rs6507708 |
| rs6567160 | .056 | C | Yes | Yes | Yes | Yes | Yes | Yes | Yes |
| rs7239883 | .015 | G | Yes | Yes | Yes | Yes | Yes | Yes | rs4289073 |
| rs7243357 | .022 | T | Yes | Yes | Yes | Yes | Yes | Yes | Yes |
| rs17724992 | .02 | A | Yes | Yes | Yes | Yes | Yes | Yes | rs34518929 |
| rs2075650 | .026 | A | Yes | Yes | Yes | Yes | Yes | Yes | rs34404554 |
| rs2287019 | .035 | C | Yes | Yes | Yes | Yes | Yes | Yes | rs11879227 |
| rs29941 | .018 | G | Yes | Yes | Yes | Yes | Yes | Yes | rs29940 |
| rs3810291 | .029 | A | Yes | Yes | rs62136856 | Yes | Yes | Yes | Yes |
| rs6091540 | .019 | C | Yes | Yes | Yes | Yes | Yes | Yes | Yes |
| rs2836754 | .017 | C | Yes | Yes | Yes | Yes | Yes | Yes | rs2836751 |

*Proxies could be found but as minor allele frequency was close to 0.5, it was not possible to determine direction

**Table S1. Two sample MR of causal effect of BMI on smoking phenotypes in the TAG consortium**

| **Method** | **Phenotype** | | | | | | | |
| --- | --- | --- | --- | --- | --- | --- | --- | --- |
|  | **Ever vs never smoking^1^**  **N =** **74,035** | | **Former vs current smoking**  **N =** **41,278** | | **Cigarettes per day**  **N =** **38,181** | | **Age at smoking initiation^2^**  **N =** **24,114** | |
|  | **OR (95% CI)** | **P** | **OR (95% CI)** | **P** | **Beta (95% CI)** | **P** | **Beta (95% CI)** | **P** |
| **IV weighted** | 1.14 (1.01, 1.29) | 0.03 | 0.97 (0.83, 1.13) | 0.68 | 1.72 (0.94, 2.51) | <0.001 | -0.01 (-0.03, 0.01) | 0.33 |
| **MR Egger slope** | 1.15 (0.84, 1.56)  1.0004 (0.992, 1.002) | 0.39  0.99 | 1.30 (0.90, 1.88)  0.992 (0.982, 1.001) | 0.16  0.09 | 1.11 (-0.86, 3.08)  0.02 (-0.03, 0.07) | 0.27  0.51 | -0.02 (-0.08, 0.03)  0.0004 (-0.001, 0.002) | 0.34  0.55 |
| **Weighted median** | 1.02 (0.84, 1.23) | 0.78 | 1.02 (0.81, 1.28) | 0.88 | 1.88 (0.71, 3.05) | 0.002 | -0.03 (-0.06, 0.01) | 0.13 |
| **Weighted mode** | 0.93 (0.69, 1.25) | 0.63 | 1.15 (0.78, 1.70) | 0.49 | 1.80 (0.19, 3.42) | 0.03 | -0.03 (-0.07, 0.01) | 0.18 |

97 SNPs used from Locke et al. BMI GWAS. Coefficients represent change in phenotype per SD increase in BMI. I-squared values for IVW were as follows: Ever vs never: 22%, p-value=0.04, Former vs current: 7%, p=0.28, CPD: 10%, p-value=0.21, Age at initiation: 9%, p-value=0.23.

1. For ever smoking, the SNP in BDNF, rs11030104 was not included in the analysis.
2. Age is log transformed so coefficients represent change in age in log units.

**Table S2. Two sample MR of causal effect of BMI on smoking phenotypes in the UK Biobank**

| **Method** | **Phenotype** | | | | | | | | | |
| --- | --- | --- | --- | --- | --- | --- | --- | --- | --- | --- |
|  | **Ever/never ^1^**  **N = 335,921** | | **Former/current**  **N =** **151,621** | | **Cigarettes per day**  **N = 100,185** | | **Age at initiation^2^**  **N = 106,239** | | **Lifetime smoking**  **N = 335,937** | |
|  | **OR (95% CI)** | **P** | **OR (95% CI)** | **P** | **Beta (95% CI)** | **P** | **Beta (95% CI)** | **P** | **Beta (95% CI)** | **P** |
| **Inverse variance weighted** | 1.21  (1.12, 1.31) | <0.001 | 0.94  (0.85, 1.03) | 0.16 | 1.34  (0.85, 1.83) | <0.001 | -0.01  (-0.02, 0.001) | 0.09 | 0.12  (0.08, 0.16) | <0.001 |
| **MR Egger slope**  **MR Egger intercept** | 1.17  (0.96, 1.43)  1.001  (0.996, 1.006) | 0.12  0.71 | 1.04  (0.83, 1.31)  1.00  (0.99, 1.00) | 0.71  0.30 | -0.32  (-1.46, 0.82)  0.05  (0.02, 0.08) | 0.58  0.002 | 0.01  (-0.02, 0.03)  -0.001  (-0.001, 0.002) | 0.49  0.13 | 0.06  (-0.04, 0.16)  0.002  (-0.001, 0.004) | 0.23  0.22 |
| **Weighted median regression** | 1.11  (1.02, 1.21) | 0.01 | 1.03 (0.90, 1.18) | 0.69 | 0.98 (0.33, 1.63) | 0.003 | -0.005  (-0.02, 0.01) | 0.53 | 0.08  (0.03, 0.12) | <0.001 |
| **Weighted mode** | 1.11  (1.00, 1.24) | 0.05 | 1.03 (0.84, 1.25) | 0.81 | 0.34 (-1.36, 2.03) | 0.70 | -0.0002  (-0.02, 0.02) | 0.99 | -0.01  (-0.07, 0.06) | 0.81 |

Using 97 BMI SNPs from Locke et al. paper. Coefficients represents change in phenotype per SD change in BMI. I-squared values for IVW were as follows: Ever vs never: 70%, Former vs current: 30%, CPD: 39%, Age at initiation: 46%, Lifetime smoking: 89%.

1. For ever smoking, the SNP in BDNF, rs11030104 was not included in the analysis.
2. Age at initiation is log transformed.

**Table S3. Two sample MR of causal effect of BMI on smoking phenotypes in females in the UK Biobank**

| **Method** | **Phenotype** | | | | | | | | | |
| --- | --- | --- | --- | --- | --- | --- | --- | --- | --- | --- |
|  | **Ever/never ^1^**  **N = 180,739** | | **Former/current**  **N =** **70,958** | | **Cigarettes per day**  **N = 47,880** | | **Age at initiation^2^**  **N = 48,021** | | **Lifetime smoking**  **N = 180,749** | |
|  | **OR (95% CI)** | **P** | **OR (95% CI)** | **P** | **Beta (95% CI)** | **P** | **Beta (95% CI)** | **P** | **Beta (95% CI)** | **P** |
| **Inverse variance weighted** | 1.23  (1.11, 1.34) | <0.001 | 0.91  (0.80, 1.03) | 0.13 | 1.34  (0.85, 1.84) | <0.001 | -0.01  (-0.02, 0.003) | 0.15 | 0.13  (0.09, 0.17) | <0.001 |
| **MR Egger slope**  **MR Egger intercept** | 1.22  (0.96 ,1.54)  1.000  (0.994, 1.004) | 0.10  0.99 | 1.03  (0.76, 1.41)  1.00  (0.99,1.00) | 0.83  0.35 | -0.06  (-1.24, 1.10)  0.04  (0.01, 0.07) | 0.91  0.01 | -0.01  (-0.04, 0.02)  -0.0001  (-0.001, 0.001) | 0.66  0.88 | 0.14  (0.05, 0.23)  -0.0002  (-0.003, 0.002) | 0.005  0.89 |
| **Weighted median regression** | 1.11  (0.99, 1.25) | 0.07 | 0.89  (0.73, 1.08) | 0.25 | 1.31  (0.58, 2.05) | <0.001 | 0.0005  (-0.02, 0.02) | 0.96 | 0.14  (0.09, 0.18) | <0.001 |
| **Weighted mode** | 1.11  (0.94, 1.32) | 0.21 | 0.92  (0.72, 1.19) | 0.53 | 1.60  (-0.33, 3.53) | 0.10 | -0.001  (-0.03, 0.03) | 0.92 | 0.05  (-0.05, 0.15) | 0.37 |

Using 97 BMI SNPs from Locke et al. paper. Coefficients represents change in phenotype per SD change in BMI. I-squared values for IVW were as follows: Ever vs never: 60%, p-value<0.001, Former vs current: 19%, p=0.05, CPD: 12%, p=0.16, Age at initiation: 12%, p=0.16, Lifetime smoking: 83%.

1. For ever smoking, the SNP in BDNF, rs11030104 was not included in the analysis.

2. Age at initiation is log transformed.

**Table S4. Two sample MR of causal effect of BMI on smoking phenotypes in males in the UK Biobank**

| **Method** | **Phenotype** | | | | | | | | | |
| --- | --- | --- | --- | --- | --- | --- | --- | --- | --- | --- |
|  | **Ever/never ^1^**  **N = 155,182** | | **Former/current**  **N =** **80,663** | | **Cigarettes per day**  **N = 52,305** | | **Age at initiation^2^**  **N = 58,218** | | **Lifetime smoking**  **N = 155,188** | |
|  | **OR (95% CI)** | **P** | **OR (95% CI)** | **P** | **Beta (95% CI)** | **P** | **Beta (95% CI)** | **P** | **Beta (95% CI)** | **P** |
| **Inverse variance weighted** | 1.21  (1.10,1.33) | <0.001 | 0.96  (0.86, 1.08) | 0.51 | 1.21  (1.10,1.33) | <0.001 | 0.96  (0.86, 1.08) | 0.51 | 0.09  (0.04, 0.14) | 0.001 |
| **MR Egger slope**  **MR Egger intercept** | 1.13  (0.90, 1.43)  1.002  (0.996, 1.008) | 0.29  0.54 | 1.06  (0.80, 1.39)  1.00  (0.99, 1.00) | 0.68  0.46 | 1.13  (0.90, 1.43)  1.002  (0.996, 1.008) | 0.29  0.54 | 1.06  (0.80, 1.39)  1.00  (0.99, 1.00) | 0.68  0.46 | -0.05  (-0.17, 0.07)  0.004  (0.001, 0.007) | 0.40  0.01 |
| **Weighted median regression** | 1.12  (1.005, 1.25) | 0.04 | 1.08  (0.90, 1.28) | 0.42 | 1.12  (1.005, 1.25) | 0.04 | 1.08  (0.90, 1.28) | 0.42 | 0.001  (-0.06, 0.06) | 0.97 |
| **Weighted mode** | 1.15  (1.00, 1.33) | 0.05 | 1.18  (0.89, 1.56) | 0.26 | 1.15  (1.00, 1.33) | 0.05 | 1.18  (0.89, 1.56) | 0.26 | -0.05  (-0.12, 0.03) | 0.21 |

Using 97 BMI SNPs from Locke et al. paper. Coefficients represents change in phenotype per SD change in BMI. I-squared values for IVW were as follows: Ever vs never: 54%, p-value<0.001, Former vs current: 10%, p=0.21, CPD: 59%, p<0.001, Age at initiation: 34%, p<0.001, Lifetime smoking: 81%.

1. For ever smoking, the SNP in BDNF, rs11030104 was not included in the analysis.

2. Age at initiation is log transformed.

**Table S5. Associations between smoking phenotypes assessed during pregnancy and *AHRR* DNA methylation in ARIES**

|  | | **AHRR** | | | | | |
| --- | --- | --- | --- | --- | --- | --- | --- |
|  | **N** | **coefficient** | **SE** | **Lower CI** | **Upper CI** | **P** | **r2** |
| **Pregnancy** |  |  |  |  |  |  |  |
| Any smoking (yes vs no) | 858 | -0.18 | 0.006 | -0.19 | -0.16 | <0.001 | 0.53 |
| Regular smoking before pregnancy (yes vs no) | 930 | -0.14 | 0.005 | -0.15 | -0.13 | <0.001 | 0.43 |
| Sustained smoking in pregnancy (yes vs no) | 826 | -0.20 | 0.006 | -0.22 | -0.19 | <0.001 | 0.58 |
| Cigs/day in 1st trimester (none, 1-9, 10+) | 933 | -0.11 | 0.004 | -0.12 | -0.11 | <0.001 | 0.47 |
| Cigs/day in 2nd trimester (none, 1-9, 10+) | 933 | -0.13 | 0.005 | -0.13 | -0.12 | <0.001 | 0.42 |
| Cigs/day in 3rd trimester (none, 1-9, 10+) | 915 | -0.12 | 0.004 | -0.12 | -0.11 | <0.001 | 0.45 |
| **Follow-up** |  |  |  |  |  |  |  |
| Current smoker (yes vs no) | 599 | -0.163 | 0.008 | -0.179 | -0.146 | <0.001 | 0.55 |
| Ever smoker (yes vs no) | 533 | -0.023 | 0.005 | -0.032 | -0.014 | <0.001 | 0.34 |

Adjusted for age and batch. Beta coefficients represent change in methylation.

**Table S6. Two sample MR of causal effect of BMI on the nicotine metabolite ratio (NMR)**

|  | **FinnTwin (N=385) 97 SNPs** | | **FINRISK (N=419) 96 SNPs** | | **YFS (N=714) 86 SNPs** | |
| --- | --- | --- | --- | --- | --- | --- |
|  | **Beta (95% CI)** | **P** | **Beta (95% CI)** | **P** | **Beta (95% CI)** | **P** |
| **Inverse variance weighted** | -1.01 (-1.68, -0.34) | 0.003 | -0.26 (-0.87, 0.36) | 0.41 | -0.27 (-0.75, 0.21) | 0.26 |
| **MR Egger slope**  **MR Egger Intercept** | -0.96 (-2.59, 0.67)  -0.002 (-0.04, 0.04) | 0.25  0.94 | 0.07 (-1.43, 1.57)  -0.009 (-0.05, 0.03) | 0.92  0.63 | -0.38 (-1.53, 0.77)  0.003 (-0.03, 0.03) | 0.51  0.83 |
| **Weighted median regression** | -1.46 (-2.45, -0.46) | 0.004 | 0.12 (-0.82, 1.07) | 0.80 | -0.18 (-0.99, 0.62) | 0.66 |
| **Weighted mode regression** | -1.81 (-3.21, -0.42) | 0.01 | 0.64 (-0.81, 2.08) | 0.39 | -0.10 (-1.05, 0.85) | 0.83 |

I-squared values from IVW were as follows: FinnTwin: I-squared=13%, p-value=0.14, FINRISK: I-squared= 0%, p-value=0.79, YFS: I-squared=0%, p-value=0.77

**Figure S1. Association of the BMI genetic risk score with BMI, stratified by smoking status**

BMI reported as kg/m^2^

**Figure S2. Scatter plots of associations between BMI SNPs and BMI in GIANT and BMI SNPs and smoking phenotypes in UK Biobank.**

A) BETA_EVER is log odds ratio for initiation, B) BETA_EX is log odds ratio for cessation, C) BETA_CPD is cigarettes per day, D) BETA_AGE is log age of initation. BETA_BMI is SD units for BMI.

**Figure S3. Scatter plots of associations between BMI SNPs and BMI in GIANT and BMI SNPs and smoking phenotypes in TAG.**

A) BETA_EVER is log odds ratio for initiation, B) BETA_EX is log odds ratio for cessation, C) BETA_CPD is cigarettes per day, D) BETA_AGE is log age of initiation. BETA_BMI is SD units for BMI.

**References**

1. Purcell S., Neale B., Todd-Brown K., Thomas L., Ferreira M. A., Bender D. et al. PLINK: a tool set for whole-genome association and population-based linkage analyses, American journal of human genetics 2007: 81: 559-575.

2. Pidsley R., CC Y. W., Volta M., Lunnon K., Mill J., Schalkwyk L. C. A data-driven approach to preprocessing Illumina 450K methylation array data, BMC genomics 2013: 14: 293.

3. Touleimat N., Tost J. Complete pipeline for Infinium (R) Human Methylation 450K BeadChip data processing using subset quantile normalization for accurate DNA methylation estimation, Epigenomics-Uk 2012: 4: 325-341.

4. Gaunt T. R., Shihab H. A., Hemani G., Min J. L., Woodward G., Lyttleton O. et al. Systematic identification of genetic influences on methylation across the human life course, Genome biology 2016: 17.

5. Houseman E. A., Accomando W. P., Koestler D. C., Christensen B. C., Marsit C. J., Nelson H. H. et al. DNA methylation arrays as surrogate measures of cell mixture distribution, Bmc Bioinformatics 2012: 13.

6. Bycroft C., Freeman C., Petkova D., Band G., Elliott L. T., Sharp K. et al. Genome-wide genetic data on ~500,000 UK Biobank participants, BioRxiv 2017.

7. O'Connell J., Sharp K., Shrine N., Wain L., Hall I., Tobin M. et al. Haplotype estimation for biobank-scale data sets, Nature genetics 2016: 48: 817-820.

8. Huang J., Howie B., McCarthy S., Memari Y., Walter K., Min J. L. et al. Improved imputation of low-frequency and rare variants using the UK10K haplotype reference panel, Nat Commun 2015: 6: 8111.

9. Howie B., Marchini J., Stephens M. Genotype imputation with thousands of genomes, G3 (Bethesda) 2011: 1: 457-470.

10. Manichaikul A., Mychaleckyj J. C., Rich S. S., Daly K., Sale M., Chen W. M. Robust relationship inference in genome-wide association studies, Bioinformatics 2010: 26: 2867-2873.
